# Single-domain flavoenzymes trigger lytic polysaccharide monooxygenases for oxidative degradation of cellulose

**DOI:** 10.1038/srep28276

**Published:** 2016-06-17

**Authors:** Sona Garajova, Yann Mathieu, Maria Rosa Beccia, Chloé Bennati-Granier, Frédéric Biaso, Mathieu Fanuel, David Ropartz, Bruno Guigliarelli, Eric Record, Hélène Rogniaux, Bernard Henrissat, Jean-Guy Berrin

**Affiliations:** 1 INRA, Aix-Marseille Université, Polytech Marseille, UMR1163 Biodiversité et Biotechnologie Fongiques, Avenue de Luminy, F-13288 Marseille, France; 2 Institute of Chemistry, Slovak Academy of Sciences, Dúbravská cesta 9, 84538 Bratislava, Slovakia; 3 Aix-Marseille Université, CNRS, UMR7281 Unité de Bioénergétique et Ingénierie des Protéines, F-13402 Marseille, France; 4 INRA, Plateforme BIBS, Unité de Recherche Biopolymères, Interactions, Assemblages, F-44316 Nantes, France; 5 CNRS, UMR7257 Architecture et Fonction des Macromolécules Biologiques, 13288 Marseille, France; 6 INRA, USC1408 Architecture et Fonction des Macromolécules Biologiques, F-13288 Marseille, France; 7 Department of Biological Sciences, King Abdulaziz University, Jeddah, Saudi Arabia

## Abstract

The enzymatic conversion of plant biomass has been recently revolutionized by the discovery of lytic polysaccharide monooxygenases (LPMOs) that carry out oxidative cleavage of polysaccharides. These very powerful enzymes are abundant in fungal saprotrophs. LPMOs require activation by electrons that can be provided by cellobiose dehydrogenases (CDHs), but as some fungi lack CDH-encoding genes, other recycling enzymes must exist. We investigated the ability of AA3_2 flavoenzymes secreted under lignocellulolytic conditions to trigger oxidative cellulose degradation by AA9 LPMOs. Among the flavoenzymes tested, we show that glucose dehydrogenase and aryl-alcohol quinone oxidoreductases are catalytically efficient electron donors for LPMOs. These single-domain flavoenzymes display redox potentials compatible with electron transfer between partners. Our findings extend the array of enzymes which regulate the oxidative degradation of cellulose by lignocellulolytic fungi.

The use of plant biomass represents an attractive alternative to fossil-based technologies for the production of high-value chemicals. In Nature, filamentous fungi produce lignocellulose-degrading enzymes to acquire carbon from plant biomass. Different types of mechanisms for the deconstruction of plant cell walls have been described in saprotrophic fungi but the involvement of oxidative enzymes was largely underestimated. The recent discovery of a new class of oxidative enzymes, namely lytic polysaccharide mono-oxygenases (LPMOs), has dramatically broadened the concept of the enzymatic deconstruction of plant cell wall polysaccharides^1^,^2^. LPMOs represent key cellulolytic enzymes that act at the surface of fibers where they mediate oxidative cleavage of the polysaccharide chains. In industry, addition of LPMOs to cellulolytic cocktails leads to the reduction of enzyme loading required for efficient saccharification of cellulosic biomass^3^.

Known LPMOs are grouped into four families of the CAZy classification^4^ (AA9, AA10, AA11 and AA13) that feature a similar histidine brace coordinating the copper ion responsible for the oxidative cleavage of the substrate^5^. LPMOs from the AA9 family are exclusively found in fungi with large expansion of genes in white-rot fungi and some ascomycetes^6^. For instance the coprophilous fungus *Podospora anserina* displays 33 genes encoding AA9 LPMOs^7^, of which 7 have been characterized biochemically^8^,^9^. They are able to oxidatively cleave cellulose and/or hemicelluloses with differences in terms of regioselectivity. LPMOs require oxygen and electrons to display activity. Oxygen is naturally available but electrons need to be supplied from reducing cofactors. LPMO activity has been demonstrated *in vitro* using ascorbate, gallate, gluthatione^10^, cysteine^11^, 3-hydroxyanthranilic acid, quinones and mixtures of low and high molecular weight fractions of lignin^12^ but the concentration of reductants used *in vitro* is generally very high. Fungal AA9 LPMOs are secreted under lignocellulolytic conditions together with cellobiose dehydrogenases (CDHs) in many fungal saprotrophs such as *Laetisaria arvalis*^13^, *Pycnoporus cinnabarinus*^14^, *Pycnoporus coccineus*^15^, *Podospora anserina*^16^ and *Neurospora crassa*^17^. CDHs are extracellular fungal flavoenzymes that harbor a flavin-binding dehydrogenase (FAD-DH) domain from the AA3_1 family and a cytochrome-domain from the AA8 family. The FAD-DH oxidizes cellobiose at C1 position to cellobionolactone by reduction of FAD, which can be re-oxidized by electron acceptors such as quinones and phenoxy radicals. Initially, several biological functions of fungal CDH were proposed in relation to the reduction of toxic quinones and lignin degradation^18^,^19^,^20^. More recently, fungal CDHs were shown to act as a reductant for LPMOs providing electrons for the redox-mediated oxidative cleavage of cellulose^8^,^21^,^22^,^23^,^24^. The electrons are rapidly transferred from the FAD to the cytochrome domain which carries a haem *b* as prosthetic group. Following the haem oxidation, the cytochrome domain of CDH reduces the copper in the active site of the AA9 LPMO to initiate oxidative cellulose breakdown^25^.

The question whether LPMOs can rely just on one enzymatic partner in the filamentous fungi has been addressed since some AA9-encoding saprotrophic fungi including *T. reesei* do not contain any genes encoding CDHs. It is likely that other enzyme redox partners apart from CDH may be secreted by fungi to trigger LPMO activity. In this study, we investigated the cooperation of AA9 LPMOs with flavin-containing enzymes from the family AA3 identified in the secretomes of lignocellulolytic fungi. The action of AA9 LPMOs in combination with several oxidative enzymes of family AA3 was evaluated on cellulose using *in vitro* assays. Based on the results of this study, a new scheme of fungal cellulose degradation in saprotrophic fungi is presented.

## Results

To find out if other redox partners than CDH could provide electrons to AA9 LPMOs, we searched for fungal flavoenzymes within the AA3_2 subfamily^6^. Potential enzyme partners were selected based on their occurrence in the secretomes of filamentous fungi upon growth under lignocellulolytic conditions. In the white-rot basidiomycete *Pycnoporus cinnabarinus* and the maize pathogen *Ustilago maydis* several AA3_2 members were identified in their secretomes upon growth under lignocellulolytic conditions^14^,^26^. These AA3_2 flavo-oxidases were heterologously produced and biochemically characterized as aryl-alcohol quinone oxidoreductases (AAQOs^27^), aryl-alcohol oxidase (AAO^28^) and glucose dehydrogenase (GDH^29^).

### Cooperation of AA9 LPMOs with aryl-alcohol quinone oxidoreductases (AAQOs) aryl-alcohol oxydase (AAO)

Three AAQOs from *P. cinnabarinus* (AAQO1, AAQO2 and AAQO3) displayed activity towards aromatic alcohols such as anisyl alcohol, 2,4-hexadien-1-ol and the lignin derivative cinnamyl alcohol. Among them, AAQO1 was considered as a strict dehydrogenase since it was unable to transfer electrons to molecular oxygen while AAQO2 and AAQO3 were considered as dehydrogenases with some residual oxidase activity^27^. On the other hand the AAO from *U. maydis* was able to efficiently oxidize anisyl alcohol using oxygen, 1,4-benzoquinone, and 2,6-dichloroindophenol as electron acceptors^28^.

**Figure 1:**
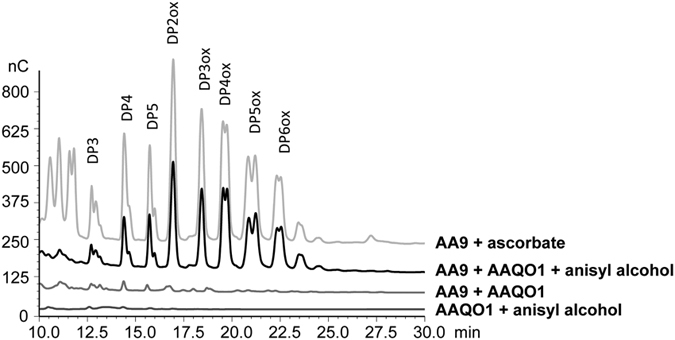
Analysis of degradation products generated by *Pa*LPMO9E in the presence of aryl-alcohol dehydrogenase (AAQO1). HPAEC chromatograms of the oligosaccharides released upon degradation of 0.1% PASC with 4.4 μM LPMO in the presence of 4.4 μM AAQO1 and 2.5 mM of anisyl alcohol, at 30 °C for 24 h. The positive control was obtained by degradation of 0.1% PASC with 4.4 μM *Pa*LPMO9E in the presence of 1 mM ascorbate, at 30 °C for 24 h.

To determine whether AAQOs and AAOs were potentiating LPMO activity, the products of cellulose degradation were analyzed by high performance anion exchange chromatography (HPAEC) under different conditions. Control reactions were carried out using the substrate and product of AAQOs and AAO, i.e. anisyl alcohol and anisyl aldehydes respectively, to make sure that these aromatic compounds were not able to transfer electrons to LPMOs (Fig. S1). In the presence of equimolar amounts of AAQO1 and *Pa*LPMO9E together with anisyl alcohol, oxidative cleavage of cellulose was observed. Among the soluble products released from cellulose, C1-oxidized oligosaccharides overlaying with the control condition (*Pa*LPMO9E with ascorbate) were detected (Fig. 1)^9^. These oxidized peaks were not present in the control conditions lacking at least one of the components of the reaction (Fig. 1). Clearly, lower amounts of degradation products were obtained when AAQO2 in the presence of its substrate coniferyl alcohol was used as a reductant for *Pa*LPMO9E (Fig. S1). No cellulose cleavage was observed when AAO was tested together with its best substrate anisyl alcohol in the presence of *Pa*LPMO9E under similar experimental conditions (Fig. S1).

**Figure 2:**
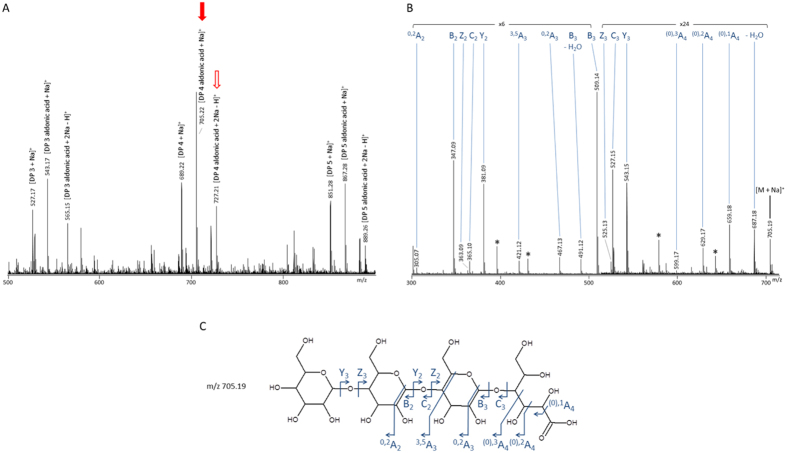
Mass spectrometry analysis of degradation products generated from PASC by AA9 LPMO with AAQO1 and anisyl alcohol as described in Fig. 1. Analyses were performed after 24 hours of cellulose degradation. Panel (**A**) shows MS spectrum of sample with peaks corresponding to native and oxidized cello-oligosaccharides. Peaks that were further fragmented are indicated by arrows. Panel (**B**) shows the MS/MS spectrum of the 705 m/z species which corresponds to a C1-oxidized product. Observed fragments are depicted on the structure in panel (**C**). Black stars: unassigned fragments.

To verify the identity of the oxidized products generated from cellulose by *Pa*LPMO9E in cooperation with AAQO1, mass spectrometry analysis of the product mixture was performed. It confirmed the presence of oxidized and non-oxidized cello-oligosaccharides. Oxidized species potentially corresponding to aldonic acid at m/z 705.22 (Δ + 16 from the non-oxidized species) which is a [M + Na]^+^ species and m/z 727.21 (Δ + 22 from the oxidized species) which is a [M + 2Na-H]^+^ species (Fig. 2A). Both MS2 fragmentation patterns of m/z 705.22 (Fig. 2B,C) and m/z 727.21 (data not shown) contain fragment ions, e.g. ^(0),1^A_4_ fragment. According to previous observation^30^, these fragments are specific of a carboxylic acid at the reducing end (C1 position) thus confirming that the oxidative cleavage of cellulose by *Pa*LPMO9E was triggered by AAQO1.

### Cooperation of AA9 LPMO with glucose dehydrogenase (GDH)

The GDH selected for synergy experiment was previously reported to be mostly active on glucose and to a lesser extent on lactose^29^. In order to find out, if products generated by GDH would not interfere with oxidative cleavage of phosphoric acid swollen cellulose (PASC), controls with GDH and cello-oligosaccharides ranging from DP1 to DP6 were performed. The analysis of products generated by GDH showed that the enzyme was able to convert cello-oligosaccharides with a degree of polymerization (DP) from DP1 to DP6 into their corresponding aldonic acids (Fig. S2). As expected, the analysis of products released from cellulose in the presence of GDH and its preferred substrate, glucose, showed only gluconic acid generated from glucose. Therefore GDH acts on glucose and cello-oligosaccharides, converting the sugars into their corresponding aldonic acids, but the enzyme does not display any observable activity towards cellulose (Fig. S2).

**Figure 3:**
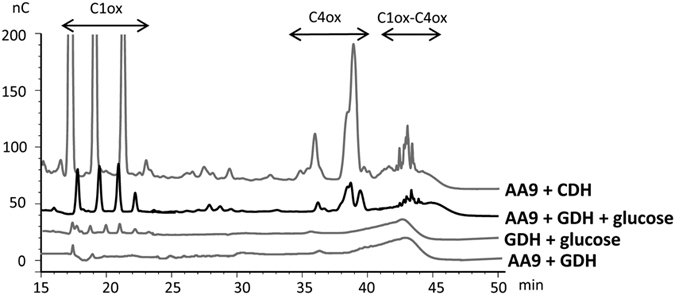
Analysis of degradation products generated from cellulose by *Pa*LPMO9H in the presence of GDH. Chromatograms of the oligosaccharides released upon degradation of 0.1% PASC with 4.4 μM LPMO in the presence of 4.4 μM GDH and 0.5 mM glucose at 40 °C for 24 h. The positive control was obtained by degradation of 0.1% PASC with 4.4 μM LPMO and 1.4 μM of CDH, at 40 °C for 24 h.

To investigate whether GDH could provide electrons to AA9 LPMOs, we decided to use the *Pa*LPMO9E and *Pa*LPMO9H from *P. anserina*, which display C1 and C1–C4 regioselectivity, respectively^9^. Combination of GDH with its substrate (glucose) and *Pa*LPMO9E did not show any difference with control reactions. However, the use of *Pa*LPMO9H resulted in the release of non-oxidized species (from DP1 to DP5), C1-oxidized species (from DP2 to DP5) and later eluted peaks, possibly corresponding to C4 and C1/C4-oxidized species (elution times 27, 35, 38 and after 41 min) (Fig. 3). These oxidized products were not detected in control reactions lacking at least one of the components of the reaction (Fig. 3).

**Figure 4:**
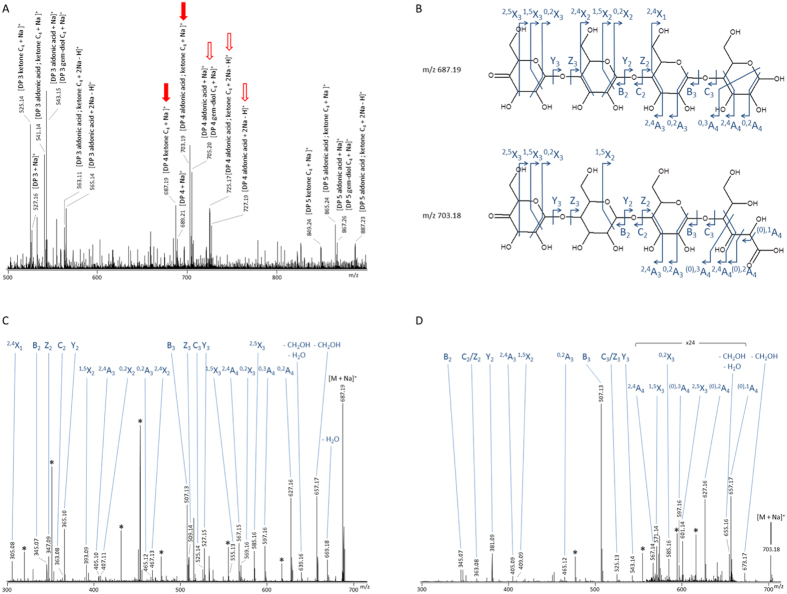
Mass spectrometry analysis of degradation products generated from PASC by *Pa*LPMO9H with GDH and glucose as described in Fig. 3. Analyses were performed after 24 hours of cellulose degradation. Panel (**A**) shows MS spectrum of sample with peaks corresponding to native and oxidized cello-oligosaccharides. Peaks that were further fragmented are indicated by arrows. Panel (**B**) shows MS/MS spectrum of the 687 m/z species which corresponds to a C4-oxidized product (ketone form). Panel (**C**) shows MS/MS spectrum of the 703 m/z species which corresponds to a double oxidized product (ketone form on C4 and aldonic acid form on C1). Observed fragments are depicted on structures in panel (**D**). Black stars: unassigned fragments.

Mass spectrometry analysis of the product mixture generated by *Pa*LPMO9H in cooperation with GDH confirmed the presence of a range of species. It included non-oxidized (*m/z* 689.21) and potentially C1-oxidized aldonic acids (*m/z* 705.20 (Δ + 16 from the non-oxidized species)), C4-oxidized species with either a ketone or a gemdiol at the non-reducing end (*m/z* 687.19 (Δ − 2 from the non-oxidized species) or *m/z* 705.20 (Δ+16 from the non-oxidized species), respectively and C1/C4 double oxidized species (*m/z* 703.19 (Δ + 14 from the non-oxidized species)) (Fig. 4). The MS2 fragmentation pattern of *m/z* 687.19 confirmed the occurrence of characteristic fragment ions (^2,5^X_3_, ^1,5^X_3_, ^1,5^X_2_) supporting the formation of a C4-oxidized ketone species (Fig. 4). On the fragmentation spectra of the *m/z* 703.19 species, fragment ions containing the reducing end (X, Y and Z fragment ions) are shifted by +16 Da compared to the ketone species at *m/z* 687.19 (Fig. 4C). On the other hand, fragment ions containing the non-reducing end (A, B and C fragment ions) are detected at the same *m/z* value as for the C4-oxidized ketone species. Altogether, this shows that the double oxidation occurs with a ketone at C4 and oxygen added at the reducing end (Fig. 4C,D). The characteristic ^(0),1^A_4_ fragment ions and the presence of an additional species at *m/z* 725.17 on the MS spectrum, corresponding to the substitution of one labile proton by a sodium, indicates the presence of a carboxylic acid at C1. These results demonstrate that the AA3_2 GDH can cooperate with LPMOs.

#### Redox potentials of flavoenzymes and LPMOs

**Figure 5:**
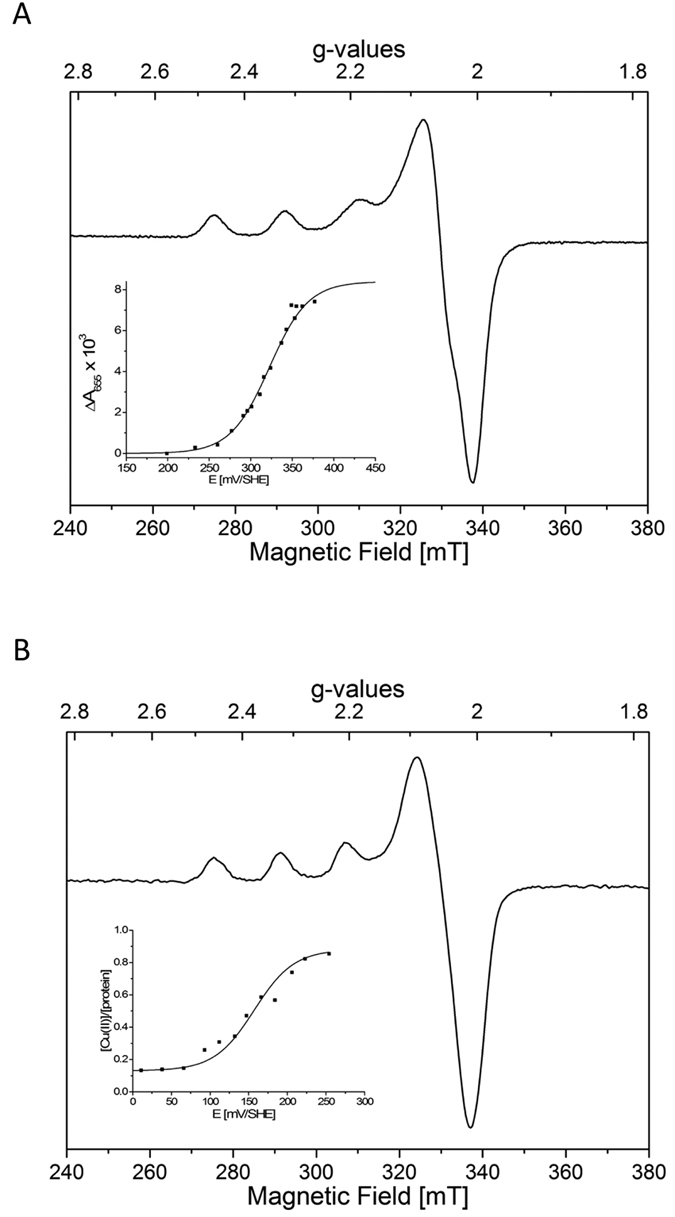
X-band EPR spectra of (**A**) *Pa*LPMO9H 300 μM and (**B**) *Pa*LPMO9E 145 μM at pH 5. Spectra were recorded at 50 K, 1 mW microwave power at 9.480 GHz, and 3 mT modulation amplitude. Titration curves are given in insets.

To better understand the interaction mechanisms of LPMO with their potential partners, redox properties of these enzymes were investigated by EPR and optical spectroscopies. Frozen solution X-band EPR spectra of *Pa*LPMO9E and *Pa*LPMO9H are typical of a near-axial mononuclear copper center (Fig. 5). Simulations of EPR spectra of *Pa*LPMO9E and *Pa*LPMO9H have been performed and estimated spin Hamiltonian parameters are summarized in Table 1. Given the lack of resolution in the high-field region, it was not possible to distinguish g_x_ and g_y_ values, and an estimation of A_x,y_^Cu^ values was not possible. The g_z_ and A_z_^Cu^ values of *Pa*LPMO9E (g_z_ = 2.263, A_z_^Cu^ = 15.7 mT) and H (g_z_ = 2.257, A_z_^Cu^ = 16.5 mT) were similar to those previously reported for AA9 enzymes^5^,^31^. Considering the usual Peisach-Blumberg classification, these values clearly place the AA9 in the type-2 copper enzymes^32^. The comparison of the Cu(II) spin Hamiltonian parameters also suggests a very similar first coordination sphere for all studied AA9. Redox titrations of *Pa*LPMO9E and *Pa*LPMO9H have been monitored by EPR and UV-visible absorption (charge transfer band at 655 nm) spectroscopies to determine the reduction potential E°′ of Cu(II) center at pH 5. The titration curves are presented in Fig. 5 (insets) and the deduced redox potential values have been reported in Table 1. Interestingly, while the redox potential of the *Pa*LPMO9H copper center (+326 mV) is in the same range as compared to those previously published for other LPMOs, the E°′ value found for *Pa*LPMO9E is clearly much lower (+155 mV). This indicates that the Cu(II)-*Pa*LPMO9H center is likely easier to reduce than that of *Pa*LPMO9E. Since the Cu(II) ion coordination sphere appears to be very similar in these enzymes, such a difference could be due to variation in the exposure of metal to the solvent and consequently, this suggests a more accessible Cu(II) center in the case of *Pa*LPMO9H.

The reduction potentials of AAQO1 and GDH flavoenzymes have also been determined for comparison with those of LPMOs. The oxidized FAD cofactor is characterized by a typical strong absorption band at 453 nm at pH 5 which disappeared when the cofactor is reduced in the FADH_2_ form. Thus, potentiometric titrations of AAQO1 and GDH have been monitored by UV-visible absorption spectroscopy and modelled by a two-electron Nernstian process to describe the FAD to FADH2 redox transition. Whereas AAQO1 has a midpoint redox potential of +86 mV, which is much lower than those of all LPMOs studied so far (Table 1), GDH is characterized by a +171 mV value which is slightly higher than that determined for *Pa*LPMO9E. One can notice that, as for the CDH cytochrome domain (Table 1), the redox potential of the FAD cofactors of AAQO1 and GDH are lower than those usually found for LPMOs, which is in agreement with a potential electron donor role to LPMOs for these flavoenzymes.

## Discussion

In this work, we showed that the combination of fungal enzymes belonging to the AA3_2 subfamily with AA9 LPMOs catalyzes the efficient cleavage of cellulose. The synergy between some AA3_2 subfamily members and AA9 LPMOs is physiologically relevant as both families are identified in fungal secretomes under lignocellulolytic conditions. *In vitro* cellulose cleavage assays were performed using equimolar amounts of enzyme partners which make the synergy even more likely to occur. Moreover, equivalent amounts of oxidized products were released in the GDH/AAQO and the ascorbate conditions but it is important to notice that the ascorbate concentration was 250-times higher than that of GDH or AAQO.

The main structural difference between CDHs and AA3_2 members is the lack of haem-binding cytochrome domain in AA3_2 s. The flavoprotein domain of CDH catalyzes the two-electrons oxidation of cellobiose^33^ using several electron acceptors such as quinones, phenoxyradicals and molecular oxygen but the latter is the least efficient^34^,^23^. Electrons from the FAD can also be transferred to the cytochrome domain which acts as a relay for LPMOs. The presence of the cytochrome domain seems to be essential for the reduction of LPMOs in order to activate the copper center^25^. In this study, we showed that the absence of a cytochrome domain in AA3_2 dehydrogenases does not seems to hamper electron transfer since efficient depolymerization of cellulose by LPMOs occurred upon GDH or AAQO1 activation. Therefore questions arise on the mechanism of electron transfer between dehydrogenases of AA3_2 subfamily and LPMOs for further understanding of the electron cascade. Another question that should be addressed is the possibility that any flavoenzyme could supply electrons to LPMOs. Our results indicate that not all AA3_2 enzymes are able to reduce LPMOs. GDH and AAQO1 are indeed strict dehydrogenases^27^,^29^ while AAQO2 displays residual oxidative activity^27^ and AAO is a strict oxidase using molecular oxygen as electron acceptor^28^. Therefore the oxygen affinity of the LPMO’s enzyme partners seems to be a key factor affecting their cooperation with LPMOs. FAD-dependent oxidases have a special binding-site for oxygen, which is not present in strict FAD-dependent dehydrogenases. The presence of bound oxygen could inhibit the interaction with LPMO or any other electron acceptor. FAD-dependent dehydrogenases have a broader spectrum of electron acceptors and this promiscuity could be beneficial for the interaction with a copper redox center as is present in LPMO.

Other factors such as the redox potentials of the different partners could play a key role in electron transfer. The redox potential for the oxidized-fully reduced free flavin is −207 mV in aqueous solution^35^. In flavoenzymes, the redox potential is strongly modulated by cofactor environment such as the presence of catalytic domain and pH. Flavo-oxidases exhibit a wide range of redox potentials from −400 mV to +90 mV. The redox potentials of the flavoenzyme AAQO1 (+86 mV) is in the same range as the redox potential of the cytochrome domain of *Neurospora crassa* CDH (+99 and +93 mV) and *Phanerochaete chrysosporium* CDH (+130 mV) but lower than the redox potentials obtained for LPMOs (between +155 and +370 mV) (Table 1). Therefore, electron transfer from AAQO1 to LPMOs is thermodynamically favored. Additionally, the higher redox potential obtained for GDH (+171 mV) is consistent with electron transfer to *Pa*LPMO9H (+326 mV). It could also explain why no collaboration was observed between GDH and *Pa*LPMO9E, which displays a much lower redox potential (+155 mV). Overall, these results indicate that redox potentials of flavo-oxidases and LPMOs need to be compatible for electron transfer. Similarly, the redox potential of low molecular reductants is an important feature of their reactivity. Indeed the fungal metabolite 3-hydroxyanthranilic acid, which has a redox potential of +242 mV (hydroxyl groups^36^) is able to reduce LPMOs^12^ while 2,2′-azino-bis-3-ethylbenzothiazoline-6-sulphonic acid (redox potential above +670 mV)^37^ and anisyl alcohol (redox potential of +1,700 mV)^37^ are not acting as reductants for LPMOs.

Exploration of the genomes of filamentous fungi showed that some fungal species with multiple AA9-encoding genes do not harbor any CDH-encoding genes (Table S1). Some of these fungal species deal with plant cell wall in their natural environment, e.g. Trichoderma species like *T. reesei*, *T. harzianum*, *T. longibrachiatum*, the ectomycorrhizae *Laccaria bicolor* and the brown-rot basidiomycete *Fomitopsis pinicola*. In the genome of these fungi, the number of AA3_2 members is quite high (between 9 and 23 genes; Table S1) suggesting the presence of potential LPMO redox partners. Interestingly, the genome of the white-rot fungi *P. cinnabarinus*, *Coprinopsis cinerea* and *Phanerochaete chrysosporium* harbor high numbers of AA9 LPMOs and AA3_2 members, some of them being co-secreted in *P. cinnabarinus*^14^. However, more biochemical characterization of AA3_2 members is necessary to strengthen their proposed function and elucidate their reactivity towards different electron acceptors.

**Figure 6:**
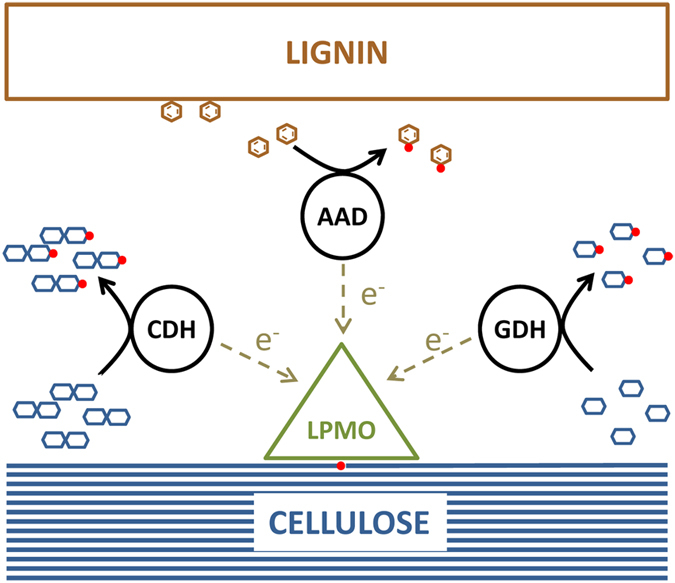
Proposed scheme of fungal synergies for oxidative degradation of cellulose. Products of cellulose and lignin degradation are substrates for fungal dehydrogenases (CDH, GDH and AAQO) which provide electrons to LPMOs.

Overall, the activation of fungal LPMOs may rely on different electrons donors among which could be CDHs, GDHs and AAQOs (Fig. 6). These enzymes are co-secreted by fungi under lignocellulolytic conditions together with cellobiohydrolases and β-glucosidases that allow the release of CDH and GDH substrates (cellobiose and glucose, respectively). Another type of mechanism may involve lignin degradation products, which are substrates for the AAQO enzyme^27^. Additionally, the ability of AAQOs, GDHs and CDHs to efficiently reduce various quinones into hydroquinones during the degradation of aromatic compounds by wood-rotting fungi, might connect the metabolism of polysaccharides and lignin by two pathways. Indeed, hydroquinones, have been recently shown to act as electron donors for LPMOs^12^.

In this work, we show for the first time cooperation between AA3_2 flavo-oxidoreductases and AA9 LPMOs for the degradation of cellulose. Our results indicate that the combinations of subtle factors such as redox potential and reactivity of dehydrogenases with molecular oxygen may play role for the cooperation between AA3 family members and AA9 LPMOs. During the reviewing process of this manuscript, two studies related to the electron sources of LPMOs have been published. Canella *et al.*^38^ have shown that light-induced electron delivery from light-harvesting pigments can efficiently drive LPMO activity. Kracher *et al.*^39^ compared different extracellular electron sources for LPMO and shown that plant-derived or fungal di-phenols/quinones serve as redox mediators between LPMO and GMC oxidoreductases thus establishing an efficient electron transfer system. Together with our study, these findings extend the array of fungal redox partners tuning oxidative degradation of cellulose in lignocellulolytic fungi. Deeper understanding of these oxidative mechanisms will be useful for the development of improved enzyme cocktails for industry.

## Material and Methods

### Production of enzymes

AA9 LPMOs and CDH from *Podospora anserina* (*Pa*LPMO9E Genbank ID CAP67740, *Pa*LPMO9H Genbank ID CAP61476 and *Pa*CDHB Genbank ID CAP61651) and AAO from *Ustilago maydis* (Genbank ID UM04044) were produced in *Pichia pastoris* in flasks or in bioreactors as described in^9^ and^28^. AAQO1 (scf185002.g8), AAQO2 (scf184746.g7) and GDH (scf184803.g17) from *P. cinnabarinus* were produced in *A. niger* as described in 27 and^29^. All of the enzymes were purified to homogeneity and quantified as described previously. For AAQO, the enzyme concentration was determined using the molar absorbance of proteins at 456 nm. These extinction coefficients were determined based on the quantification of free FAD extracted from the protein samples by heat denaturation and using the equation: ε456 = εFAD × A456/A450, where A456 is the enzyme absorbance at 456 nm, A450 is the free-FAD absorbance at 450 nm, and εFAD is the free-FAD extinction coefficient at 450 nm (11300 M^−1^ cm^−1^). The estimated extinction coefficients for the purified AAQO1 and AAQO2 were ε456 = 11,300 M^−1^ cm^−1^ and ε456 = 10,500 M^−1^ cm^−1^, respectively. The A280/A456 ratios were found to be 10 for AAQO1 and 12.5 for AAQO2 indicating that the proteins were in their holoforms.

### Cellulose cleavage assays

All the cleavage assays (300 μL liquid volume) contained 4.4 μM of AA9 LPMOs, and equimolar concentration of AAO, AAQO1, AAQO2 or GDH, 2.5 mM anisyl or coniferyl alcohol and 0.5 mM of glucose or cello-oligosaccharides, respectively and 0.1% (w/v) PASC prepared from Avicel as described by^40^ in 50 mM sodium acetate phosphate buffer pH 5. As a positive control, reaction mixtures of AA9 LPMO with 1 mM of ascorbate or 1.4 μM of *Pa*CDHB and 0.1% (w/v) PASC were prepared as described previously^9^. The enzyme reactions were performed in 2-mL tubes and incubated in a thermomixer (Eppendorf) at 30 °C and 850 rpm. After 24 h of incubation, samples were boiled at 100 °C for 10 min to stop the enzymatic reaction and then centrifuged at 16,000 × g for 15 min at 4 °C to separate the soluble fraction from the remaining insoluble fraction before carbohydrate determination. The soluble products were analyzed by high-performance anion exchange chromatography as described by^41^.

### Analysis of oxidized products using mass spectrometry

Products resulting from enzyme reaction in water as described above were analyzed by ESI-MS (Electrospray ionization). Experiments were performed on a Synapt G2Si high-definition mass spectrometer (Waters Corp., Manchester, UK). Two types of mass measurements were performed on the samples: firstly, a mass profile was done on a mass range of 500–2000 m/z. Ions of interest were further fragmented by collision-induced in the transfer cell of the instrument, using appropriate collision energies depending on the precursor. Samples were diluted 10-fold in MeOH/H_2_O (1:1, v/v) and infused at 5 μL.min^−1^ in the instrument. The instrument was operated in a positive ionization mode in the so-called sensitivity mode, with an ESI capillary voltage of 3 kV and a sampling cone voltage of 100 V. Data acquisition was carried out using MassLynx software (V4.1).

### EPR spectrometry

EPR measurements were performed on a Bruker ELEXSYS E500 spectrometer. X-band EPR spectra were recorded using a standard rectangular Bruker cavity (ST) fitted to an Oxford Instruments ESR900 helium flow cryostat.

### Redox titration experiments

Midpoint redox potentials of AA9 LPMOs, AAQO and GDH were determined by potentiometric titration at pH 5 in 0.1 M acetate buffer followed by EPR or UV-Vis absorption spectroscopies. Titrations were performed at room temperature in an anaerobic glovebox. Redox potentials were adjusted with small additions of 8 mM sodium dithionite and measured with a combined Pt-Ag/AgCl/KCl (3 M) Mettler-Toledo micro-electrode calibrated by using redox buffer solutions and are given in the text with respect to the standard hydrogen electrode. The following redox mediators were used at 10 μM final concentrations for all titrations: 1,1′ ferrocene dimethanol; ferrocene; N,N dimethyl-p-phenyldiamine; 1,4-benzoquinone; dichlorophenolindophenol; 1,2-naphtoquinone; phenazine ethosulfate. For LPMOs titration, mono carboxylic acid ferrocene was also added as mediator. For flavoenzymes titration, duroquinone, 1,4-dihydroxynaphtoquinone, indigo carmine and anthraquinone-2,6-disulfonate were also added as mediators. For EPR spectroscopy, samples were anaerobically transferred into calibrated EPR tubes that were rapidly frozen in glove box.

## Additional Information

**How to cite this article**: Garajova, S. *et al.* Single-domain flavoenzymes trigger lytic polysaccharide monooxygenases for oxidative degradation of cellulose. *Sci. Rep.* **6**, 28276; doi: 10.1038/srep28276 (2016).

## Supplementary Material

Supplementary Information

## Figures and Tables

**Table 1:** Redox potentials and spin Hamiltonian parameters for LPMOs and flavoenzymes.

	E°′ [mV/SHE]	g_x_	g_y_	g_z_	A_z_^Cu^ [10^−4^ cm^−1^]	reference
*Pa*AA9E	+155 (pH 5)	2.054	2.054	2.263	147	this work
*Pa*AA9H	+326 (pH 5)	2.052	2.052	2.257	154	this work
*Ta*AA9A	n.d.	2.06	2.06	2.27	153	5
*Nc*AA9C	+224	n.d.	n.d.	2.267	152	31
*Ba*AA10A	+275/+370	n.d.	n.d.	2.25	135	42
*Bl*AA10A	n.d.	2.038	2.108	2.262	125	43
*Sm*AA10A	n.d.	2.039	2.116	2.260	116	43
*Sc*AA10B	+251	2.020	2.090	2.270	158	43
*Tf*AA10B	n.d.	2.018	2.103	2.262	156	43
*Sc*AA10C	+242	2.015	2.102	2.267	153	43
*Sm*AA10A	+275	n.d.	n.d.	n.d.	n.d.	44
*Ao*AA11	n.d.	2.03	2.10	2.28	147	45
*An*AA13	n.d.	2.047	2.077	2.259	151	11
AAQO1 (FAD)	+86 (pH 5)					this work
GDH (FAD)	+171 (pH 5)					this work
*Tv*CDH (FAD) *Tv*CDH (haem)	−163 (pH 3) −40 (pH 3)					46
*Nc*CDHIIA (haem)	+99 (pH 6)					23
*Nc*CDHIIB (haem)	+93 (pH 6)					23
*Pc*CDH (haem)	+130 (pH 7)					47
