# Supplementary material for: Single-domain flavoenzymes trigger lytic polysaccharide monooxygenases for oxidative degradation of cellulose
Source: Sci Rep. 2016 Jun 17;6:28276. doi: 10.1038/srep28276 (PMC4911613; doi:10.1038/srep28276)
Supplement: Supplementary Information [file srep28276-s1.pdf]

Supplementary information

Single-domain flavoenzymes trigger lytic polysaccharide monooxygenases for oxidative degradation of cellulose

Sona Garajova[1,2], Yann Mathieu[1], Maria Rosa Beccia[3], Chloé Bennati-Granier[1], Frédéric Biaso[3], Mathieu Fanuel[4], David Ropartz[4], Bruno Guigliarelli[3], Eric Record[1], Hélène Rogniaux[4], Bernard Henrissat[5,6,7], Jean-Guy Berrin[1,*]

Figure S1. (A) HPAEC chromatograms of the products released upon degradation of 0.1% PASC in the presence of 4.4 μM AA9 LPMO and 2.5 mM anisyl aldehyde or 2.5 mM anisyl alcohol, at 30°C for 24 h. (B) HPAEC chromatograms of the products released upon degradation of 0.1% PASC in the presence AAQO2 or AAO with or without their substrates 2.5 mM coniferyl alcohol and anisyl alcohol, respectively, at 30°C for 24 h. The peak annotations are based on comparison with oligosaccharides standards oxidized at the C1 position (DP2ox-DP5 ox). Coelution of DP1ox with DP3 and DP2ox with DP6 was observed.

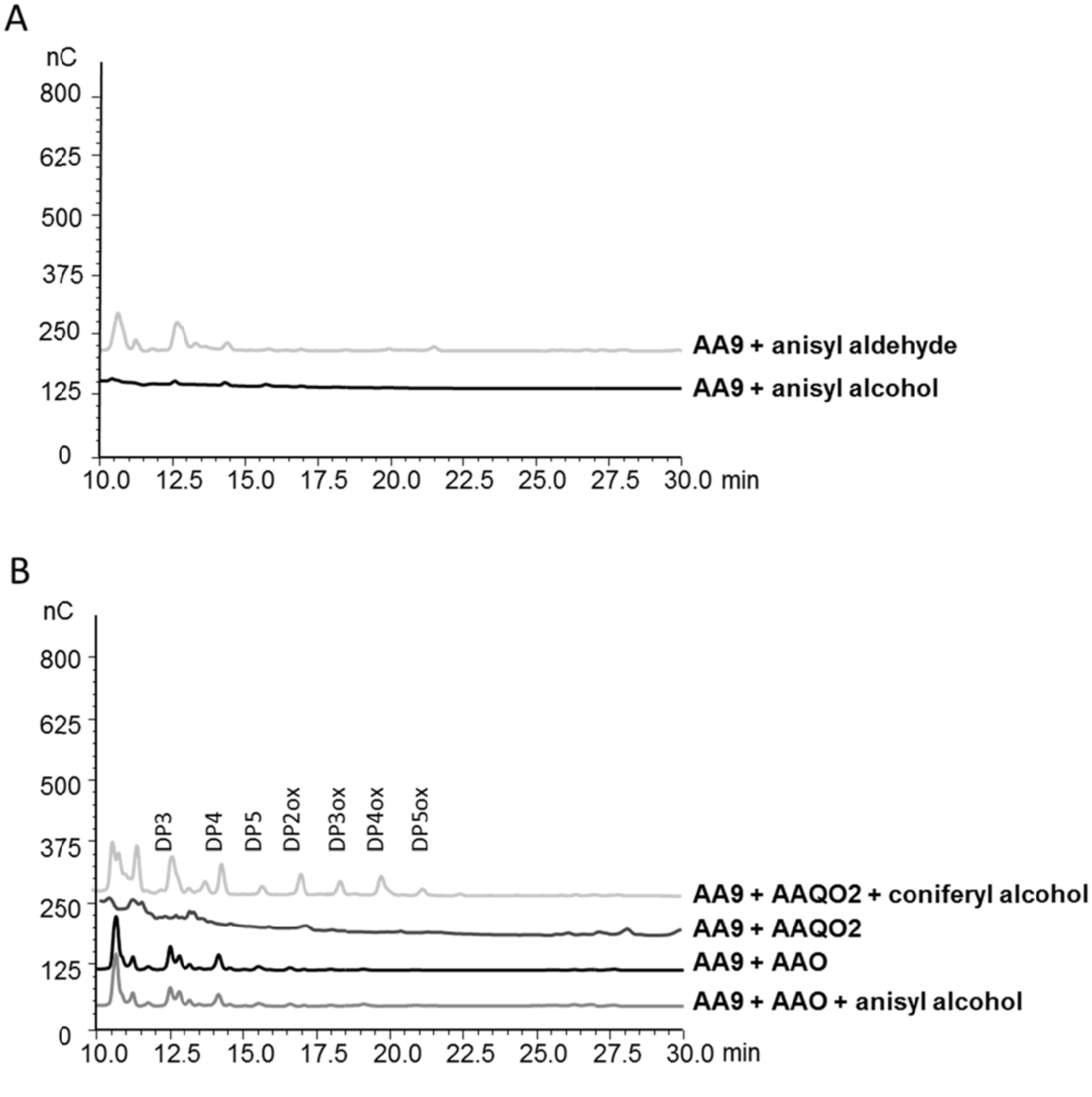

Figure S2. Chromatograms of the products released upon degradation of 0.1% PASC in the presence of 4.4 μM GDH and 0.5 mM cellooligosaccharides with DP1-DP6. The peak annotations are based on comparison with oligosaccharides standards oxidized at the C1 position (DP2ox-DP5 ox). Coelution of DP1ox with DP3 and DP2ox with DP6 was observed.

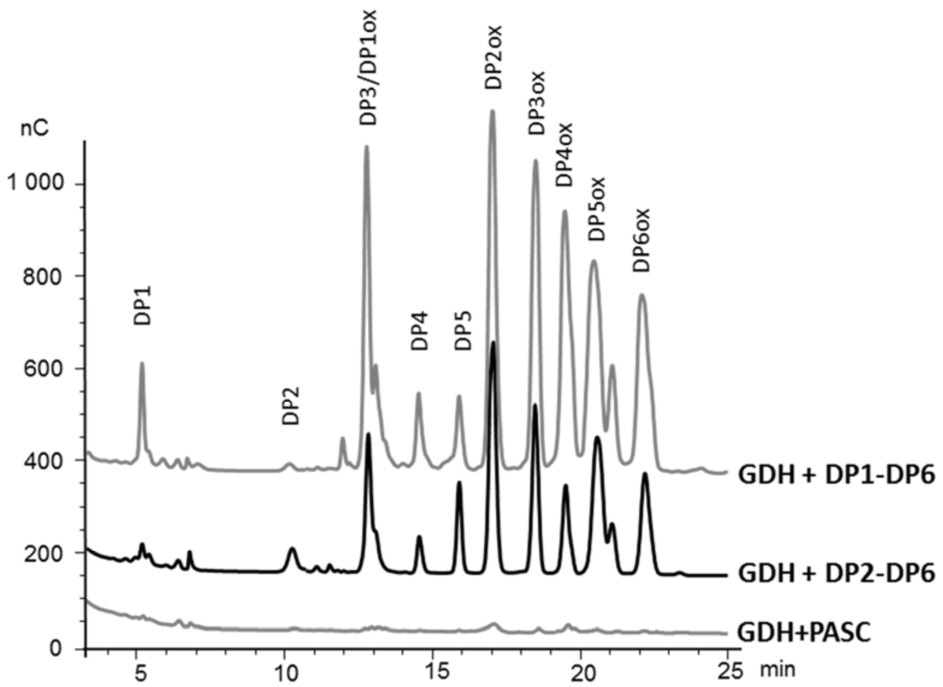

Table S1. Number of AA3_1, AA3_2 and AA9 LPMO genes in some fungal genomes.

Fungal species	AA3_1	AA3_2	AA9
Trichoderma harzianum	0	16	3
Trichoderma virens	0	14	3
Trichoderma reesei	0	10	3
Trichoderma citrinoviride	0	10	3
Trichoderma longibrachiatum	0	9	3
Trichoderma atroviride	0	9	3
Rozella allomycis	0	10	3
Rhodosporidium toruloides	0	10	3
Rhodotorula graminis	0	11	3
Microbotryum violaceum	0	11	3
Neolentinus lepideus	0	23	4
Fomitopsis pinicola	0	16	4
Daedalea quercina	0	13	4
Laccaria bicolor	0	13	4
Talaromyces aculeatus	0	21	5
Amanita muscaria	0	6	2
Tuber melanosporum	1	4	2
Zymoseptoria tritici	1	13	2
Penicillium chrysogenum	1	19	4
Ceriporiopsis subvermispora	1	17	9
Aspergillus nidulans	1	22	10
Heterobasidion annosum	1	29	10
Agaricus bisporus	1	29	11
Ganoderma lucidum	1	19	15
Phanerochaete chrysosporium	1	31	15
Pycnoporus cinnabarinus	1	20	17
Trametes versicolor	1	17	18
Schizophyllum commune	1	22	22
Pleurotus eryngii	1	29	31
Coprinopsis cinerea	1	35	34
Aspergillus clavatus	2	15	7
Nectria haematococca	3	18	12
Thielavia terrestris	3	7	19
Podospora anserina	3	18	33
Fusarium verticillioides	5	16	13
Fusarium oxysporum	5	17	16
Magnaporthe grisea	5	11	24
